# Associations Between Body Composition and Bone Mineral Density in Adults with Metabolic Syndrome: A DEXA-Based Cross-sectional Study

**DOI:** 10.5812/ijem-170210

**Published:** 2026-04-26

**Authors:** Ngo Thi Thu Thuy, Nguyen Ngoc Chau, Pham Van Thuc, Le Van Thieu, Nguyen Thi Van Khanh, Tran Vuong The Vinh

**Affiliations:** 1 Department of Internal Medicine, Hai Phong Medical University Hopspital, Hai Phong, Vietnam; 2 Department of Rheumatology, 108 Military Central Hospital, Hanoi, Vietnam; 3 Department of Pathophysiology, Clinical Allergy and Immunology, Hai Phong University of Medicine and Pharmacy, Hai Phong, Vietnam; 4 Department of Endoscopy and Functional Exploration, Viet Tiep Friendship Hospital, Hai Phong, Vietnam; 5 Department of Internal Medicine, Can Tho University of Medicine and Pharmacy, Can Tho, Vietnam; 6 Department of Surgery, Hai Phong University of Medicine and Pharmacy, Hai Phong, Vietnam

**Keywords:** Body Composition, Bone Mineral Density, Lean Mass, Metabolic Syndrome, Fat Mass

## Abstract

**Background and Objective:**

Body composition is increasingly recognized as an important factor associated with bone health; however, evidence focusing on individuals with metabolic syndrome (MetS) remains limited, particularly in Southeast Asian populations.

**Methods:**

This cross-sectional study included 128 adults with MetS recruited from two medical centers in Vietnam between October 2017 and September 2024. All participants underwent dual-energy X-ray absorptiometry (DEXA) to assess regional body composition and bone mineral density (BMD). Associations between lean mass, fat distribution, and BMD across multiple skeletal sites were evaluated using Pearson correlation analysis.

**Results:**

Lean mass indices, including Lean Mass Index (LMI) and Appendicular Skeletal Muscle Index (ASMI), were positively associated with BMD across multiple skeletal sites, with the strongest associations observed in the appendicular skeleton and whole body (r = 0.17 - 0.73, P < 0.001). In contrast, central adiposity, reflected by the android-to-gynoid (A/G) fat ratio and regional fat percentages, showed inverse associations with BMD, particularly at the spine and long bones (r = -0.20 to -0.29, P < 0.05).

**Conclusions:**

In adults with MetS, lean mass was positively associated with BMD, whereas central adiposity showed inverse associations with skeletal health. These findings highlight the importance of body composition, beyond BMI alone, in the assessment of bone health in this high-risk population. DEXA-based body composition assessment may provide clinically relevant information to support patient evaluation.

## 1. Background

Metabolic syndrome (MetS) has become increasingly prevalent worldwide and is a major risk factor for cardiovascular disease and type 2 diabetes mellitus. Beyond these well-established associations, MetS also exerts profound effects on musculoskeletal health. Altered body composition, including reduced lean mass and excessive visceral adiposity, may be associated with bone mineral density (BMD) (1, 2).

Traditionally, greater body weight was believed to protect bone through mechanical loading. However, accumulating evidence suggests that the benefits of lean muscle mass and the detrimental effects of central adiposity should be considered separately. Lean mass has been associated with skeletal strength, rehabilitation potential, and functional recovery, while excess visceral fat promotes chronic inflammation, impairs bone metabolism, and increases the risk of fragility fractures and delayed healing. Regional fat distribution, particularly the android-to-gynoid (A/G) ratio, has emerged as a more precise indicator of skeletal vulnerability and potential clinical risk than Body Mass Index (BMI) (3-10).

Most studies investigating these relationships have been conducted in Western populations, where body composition differs significantly from that of Asians. At a given BMI, Asian individuals tend to have higher visceral fat and lower lean mass, predisposing them to both metabolic and skeletal fragility. Data from Southeast Asia remain limited regarding how body composition relates to skeletal health and clinical risk assessment (11).

In Vietnam, the prevalence of MetS is rising rapidly due to urbanization, aging, and lifestyle changes (12). Yet, no study has comprehensively evaluated the associations between body composition, fat distribution, and BMD in Vietnamese adults using dual-energy X-ray absorptiometry (DEXA), the gold standard for these assessments. Addressing this gap is clinically important for metabolic and musculoskeletal risk assessment in adults with MetS (13).

## 2. Objectives

This study aimed to examine the associations between BMD and regional body composition parameters, including lean mass indices and fat distribution, in Vietnamese adults aged 40 years or older with MetS. By integrating DEXA-derived parameters with metabolic and anthropometric data, this study may provide clinically relevant insights into metabolic and musculoskeletal risk assessment in adults with MetS.

## 3. Methods

### 3.1. Study Design and Subjects

This was a cross-sectional study conducted between October 2017 and September 2024 at two medical centers. The research was approved by Hai Phong University of Medicine and Pharmacy Institutional Review Board and an ethics committee (IRB No. 5/HDDD). All participant information was kept confidential in accordance with ethical guidelines. The study enrolled adults aged 40 years or older who were diagnosed with MetS according to the 2009 Joint Interim Statement criteria. All analyses in the present study focused exclusively on individuals with MetS. Adults aged 40 years or older were selected because this age range represents a period during which both metabolic abnormalities and age-related decline in bone health become increasingly prevalent, making it a clinically relevant group for examining the interaction between body composition and skeletal status.

Participants were recruited from adults attending the participating medical centers during the study period. Individuals were screened for eligibility based on clinical evaluation, anthropometric measurements, laboratory findings, and DEXA availability. Recruitment was hospital-based and followed a consecutive sampling approach among eligible adults with MetS. The final analytic sample included 128 participants who met all inclusion criteria and none of the exclusion criteria.

Eligibility, comorbid conditions, medication use, and exclusion criteria were assessed through a combination of medical record review, participant interview, and physician evaluation at the time of enrollment.

### 3.2. Inclusion Criteria

- Age: Adults aged 40 years or older at the time of recruitment.

- General health status: Able to walk independently or with minimal assistance and engage in routine daily activities.

- Consent: Willing and able to provide written informed consent after being fully informed about the nature, objectives, and procedures of the study. Diagnosed with MetS based on the 2009 Joint Interim Statement criteria (IDF/AHA/NHLBI/WHO), requiring the presence of at least three of the following components: a. Abdominal obesity: waist circumference ≥ 90 cm in men or ≥ 80 cm in women (Asian cut-off) b. Elevated triglycerides: ≥150 mg/dL or current treatment for hypertriglyceridemia c. Reduced HDL cholesterol: < 40 mg/dL in men or < 50 mg/dL in women d. Elevated blood pressure: systolic BP ≥ 130 mmHg and/or diastolic BP ≥ 85 mmHg, or use of antihypertensive medication e. Elevated fasting plasma glucose: ≥ 100 mg/dL or previously diagnosed type 2 diabetes

### 3.3. Exclusion Criteria

Participants were excluded if any of the following criteria were present: (1) Autoimmune diseases, (2) Cirrhosis or renal failure , (3) Tuberculosis or currently undergoing TB treatment, (4) Chronic arthritis, hyperthyroidism, primary hyperparathyroidism, Cushing’s syndrome (5) Chronic liver or kidney disease, multiple myeloma, (6) Prolonged immobility, (7) Receiving hormone replacement therapy, (8) History of oophorectomy, (9) Current or long-term use of corticosteroids, (10) History of fracture, vertebral compression, or orthopedic surgery, (11) Incomplete data collection, (12) Currently taking medications affecting BMD: diuretics, bisphosphonates, selective estrogen receptor modulators, hormone replacement therapy, glucocorticosteroids, calcitonin, anticonvulsants (e.g., phenytoin, phenobarbital, carbamazepine), anticoagulants (e.g., heparin), methotrexate, (13) Medications with known or potential effects on bone metabolism were excluded to reduce confounding in the analysis of BMD. This exclusion was applied because some subclasses may affect calcium handling and bone turnover.

### 3.4. Sample Size Calculation

Based on a previous study examining correlations between body composition and BMD, a minimum sample size of 40 participants was considered sufficient to detect a moderate correlation coefficient (r ≥ 0.40) with 80% statistical power at a two-sided significance level of 0.05.14 The final sample size of 128 participants exceeded this requirement, providing adequate power for correlation analyses.

### 3.5. Anthropometric and Clinical Measurements

All anthropometric measurements were performed by trained medical staff in accordance with standardized procedures. Participants wore light clothing and were barefoot during the assessment.

Standing height was determined using a fixed stadiometer and recorded to the nearest 0.1 cm. Subjects stood in an upright posture with their heels together and head aligned in the Frankfurt plane. Body weight was measured using a calibrated electronic scale and recorded to the nearest 0.1 kg. The device was checked and zeroed prior to each measurement. BMI was subsequently calculated as body weight divided by the square of height and expressed in kg/m².

Waist circumference was assessed at the midpoint between the lower costal margin and the iliac crest using a flexible measuring tape, whereas hip circumference was measured at the level of the greatest posterior protuberance of the buttocks. Both measurements were obtained at the end of gentle expiration with the participant standing naturally and arms at rest. The waist-to-hip ratio was calculated as the ratio of waist circumference to hip circumference.

Central fat distribution was further evaluated using the android-to-gynoid (A/G) fat ratio derived from regional fat mass measurements obtained by dual-energy X-ray absorptiometry (DEXA), as described below.

Blood pressure was measured using an automated, validated device with the participant in a seated position after a rest period of at least five minutes. Two measurements were taken from the right arm with an interval of approximately two minutes, and the mean value was used for analysis. Participants were instructed to refrain from caffeine intake, smoking, and physical activity for at least 30 minutes prior to the assessment.

### 3.6. Laboratory Tests

Venous blood specimens were collected from all participants in the morning between 7:00 and 9:00 AM following an overnight fast of at least 8–12 hours. Sample collection was performed by trained laboratory personnel under aseptic conditions using EDTA-containing and serum-separation tubes.

Biochemical analyses, including fasting plasma glucose, triglycerides (TG), high-density lipoprotein cholesterol (HDL-C), and other routine parameters, were conducted at the central laboratory using automated enzymatic colorimetric techniques. Laboratory quality assurance was maintained through daily internal control procedures in compliance with ISO 15189 accreditation standards.

Circulating leptin and adiponectin levels were measured using commercially available enzyme-linked immunosorbent assay kits (Human Leptin ELISA and Human Adiponectin ELISA; BioVendor, Czech Republic). All measurements were performed in duplicate in accordance with the manufacturer’s protocols. The coefficients of variation for intra-assay and inter-assay precision were less than 8% and 10%, respectively.

Following collection, blood samples were centrifuged at 3000 rpm for 10 minutes at 4°C. The separated serum was aliquoted and preserved at -80°C until further analysis. All assays were performed in the same certified laboratory to ensure consistency and reduce analytical variability.

### 3.7. Bone Mineral Density and Body Composition Assessment

Bone mineral density and body composition were evaluated using whole-body DEXA with a Hologic Discovery QDR system (Hologic Inc., Bedford, MA, USA). The instrument was calibrated each day with standard phantoms provided by the manufacturer to maintain measurement precision. Bone mineral density values were expressed in g/cm², and T-scores were calculated using the Asian reference database incorporated in the DEXA system.

All scans were conducted by trained and certified technicians following established protocols. Participants were examined in the supine position and were asked to remain motionless throughout the scanning procedure while wearing light clothing.

Bone density measurements were obtained for predefined anatomical regions, including the whole body, lumbar spine (L1–L4), pelvis, and bilateral upper and lower limbs.

The DEXA system also provided detailed body composition parameters, including total and regional fat mass and lean mass. Regional fat distribution was further characterized by automated estimation of android and gynoid fat compartments, from which the android-to-gynoid (A/G) fat ratio was calculated.

To account for individual differences in body size, normalized indices were computed. The Fat Mass Index (FMI) was defined as total fat mass divided by height squared (kg/m²), and the Lean Mass Index (LMI) was calculated using the same approach for lean mass.

All scans were subsequently reviewed by an experienced clinician specializing in densitometry to ensure data quality and accuracy.

### 3.8. Statistical Analysis

All statistical analyses were performed using SPSS software (version 26.0; IBM Corp., Armonk, NY, USA). Continuous variables are presented as mean ± standard deviation (SD). The distribution of continuous variables was assessed using the Shapiro–Wilk test. Pearson correlation coefficients were used to evaluate the strength and direction of associations between BMD and body composition variables, including lean mass indices and regional fat distribution measures.

Given the exploratory nature of the study, Pearson correlation analysis was selected to characterize overall association patterns across multiple skeletal sites. No partial correlation or multivariable linear regression models were applied. This decision was based on the moderate sample size, the strong intercorrelation among body composition variables (raising concerns about multicollinearity), and the absence of several clinically relevant covariates, including menopausal status, physical activity, calcium intake, and vitamin D status. Because multiple correlations were examined across several anatomical regions, no formal correction for multiple comparisons was performed. Therefore, the results should be interpreted with caution, particularly for associations of smaller magnitude. A two-sided P-value < 0.05 was considered statistically significant.

## 4. Result

### 4.1. Patient Characteristics

A total of 128 adults with MetS were included. The mean age of the participants was 59.99 ± 6.59 years, and the majority were female (80.5%), while males accounted for 19.5% of the study population. The mean BMI was 23.2 ± 2.6 kg/m². More than half of the participants (53.1%) were classified as overweight or obese according to Asian BMI criteria (BMI > 23 kg/m²), whereas 42.2% had a normal BMI (18.5 - < 23 kg/m²) and only 4.7% were underweight (BMI < 18.5 kg/m²). The mean body weight was 57.43 ± 8.79 kg, and the mean height was 157.56 ± 7.75 cm. Indicators of central obesity were prominent in this cohort. The mean waist circumference was 84.76 ± 7.10 cm, and the mean hip circumference was 92.28 ± 5.67 cm. The median waist-to-hip ratio (WHR) was 0.92 (interquartile range: 0.89 - 0.95), reflecting a high prevalence of abdominal fat accumulation among individuals with MetS. The study population was predominantly female (80.5%). Because of the relatively small number of male participants, sex-stratified analyses were not performed.

### 4.2. Correlation Between Bone Mineral Density and Regional Lean Mass

Bone mineral density showed consistent and significant positive correlations with lean mass across nearly all skeletal sites. The strongest associations were observed in long bones, particularly the arms (e.g., left arm BMD and gynoid lean mass: r = 0.73; right arm and trunk lean mass: r = 0.63; both P < 0.001), and legs (e.g., left leg and trunk lean mass: r = 0.61; right leg and gynoid lean mass: r = 0.63). Axial sites such as the spine and pelvis also showed moderate-to-strong correlations with trunk, android, and LMI values (r > 0.5). The LMI and Appendicular Skeletal Muscle Index (ASMI) were positively correlated with whole-body BMD (r = 0.60 for both) and T-score (r = 0.38 and 0.31, respectively). These findings suggest that lean mass, particularly in central and appendicular regions, was consistently associated with higher BMD (Table 1). 

**Table 1.** Pearson Correlation Coefficients (r) Between Bone Mineral Density and Lean Mass Parameters ^a^

Region and BMD site	Arm	Trunk	Leg	Whole Body	Android	Gynoid	LMI	ASMI
**Axial skeleton**								
Skull	0.24 ^b^	0.36 ^c^	0.30 ^c^	0.35 ^c^	0.25 ^b^	0.37 ^c^	0.19	0.17
Spine	0.39 ^c^	0.57 ^c^	0.54 ^c^	0.59 ^c^	0.50 ^c^	0.62 ^c^	0.41 ^c^	0.36 ^c^
Rib – left	0.31 ^c^	0.54 ^c^	0.35 ^c^	0.48 ^c^	0.37 ^c^	0.43 ^c^	0.35 ^c^	0.23 ^b^
Rib – right	0.32 ^c^	0.55 ^c^	0.34 ^c^	0.48 ^c^	0.42 ^c^	0.44 ^c^	0.41 ^c^	0.26 ^b^
**Appendicular skeleton**								
Arm – left	0.58 ^c^	0.65 ^c^	0.59 ^c^	0.54 ^c^	0.64 ^c^	0.73 ^c^	0.54 ^c^	0.69 ^c^
Arm – right	0.46 ^c^	0.63 ^c^	0.58 ^c^	0.65 ^c^	0.50 ^c^	0.63 ^c^	0.48 ^c^	0.40 ^c^
Leg – left	0.47 ^c^	0.61 ^c^	0.59 ^c^	0.65 ^c^	0.49 ^c^	0.65 ^c^	0.50 ^c^	0.43 ^c^
Leg – right	0.46 ^c^	0.61 ^c^	0.57 ^c^	0.64 ^c^	0.48 ^c^	0.63 ^c^	0.49 ^c^	0.42 ^c^
**Axial/central**								
Pelvis	0.41 ^c^	0.62 ^c^	0.44 ^c^	0.58 ^c^	0.48 ^c^	0.57 ^c^	0.45 ^c^	0.31 ^c^
**Whole body**								
Total body	0.44 ^c^	0.58 ^c^	0.52 ^c^	0.60 ^c^	0.44 ^c^	0.61 ^c^	0.44 ^c^	0.60 ^c^
T-score	0.37 ^c^	0.51 ^c^	0.45 ^c^	0.54 ^c^	0.37 ^c^	0.54 ^c^	0.38 ^c^	0.31 ^c^

Abbreviations: ASMI, Appendicular Skeletal Muscle Index; LMI, Lean Mass Index; BMD, bone mineral density.

^a^ Values are Pearson correlation coefficients (r). All coefficients were tested statistically using two-sided P-values.

^b^ P < 0.05.

^c^ P < 0.01.

### 4.3. Correlation Between Bone Mineral Density and Regional Fat Percentage

Higher fat percentage, particularly in central regions, was negatively correlated with BMD at several skeletal sites. The spine exhibited the most consistent inverse associations (e.g., android fat %: r = -0.29; gynoid fat %: r = -0.28; leg fat %: r = -0.20 to -0.23, all P < 0.05). Significant negative correlations were also observed in long bones, such as the arms (e.g., right arm BMD and android fat %: r = -0.24) and legs (e.g., left leg and leg fat %: r = -0.24; right leg: r = -0.23). In contrast, no significant associations were found between fat percentage and BMD in flat bones such as the skull or ribs. The T-score showed weak, non-significant inverse correlations with all fat regions. These results highlight a detrimental association between regional fat accumulation, especially central adiposity, and skeletal health in MetS patients (Table 2). 

**Table 2.** Pearson Correlation Coefficients (r) Between Bone Mineral Density and Regional Fat Percentage ^a^

Region and BMD site	Arm	Trunk	Leg	Whole Body	Android	Gynoid	Leg – L	Leg – R	Pelvis
**Axial skeleton**									
Skull	-0.01	0.02	-0.004	-0.01	-0.02	-0.03	-0.06	-0.10	-0.09
Spine	-0.23 ^b^	-0.15	-0.12	-0.15	-0.29 ^c^	-0.28 ^c^	-0.20 ^b^	-0.23 ^b^	-0.30 ^c^
Rib – left	-0.08	0.04	0.10	0.05	0.02	0.01	0.04	0.004	-0.01
Rib – right	-0.10	-0.07	-0.01	0.01	-0.04	-0.04	-0.03	0.004	-0.02
**Appendicular skeleton**									
Arm – left	-0.18	-0.15	-0.16	-0.16	-0.23 ^b^	-0.22 ^b^	-0.18	-0.15	-0.21 ^b^
Arm – right	-0.26 ^c^	-0.15	-0.17	-0.20 ^b^	-0.24 ^b^	-0.24 ^b^	-0.21 ^b^	-0.22 ^b^	-0.26 ^c^
Leg – left	-0.32 ^c^	-0.24 ^b^	-0.21 ^b^	-0.23 ^b^	-0.20 ^b^	-0.21 ^b^	-0.24 ^b^	-0.23 ^b^	-0.24 ^b^
Leg – right	-0.30 ^c^	-0.23 ^b^	-0.20 ^b^	-0.21 ^b^	-0.20 ^b^	-0.21 ^b^	-0.23 ^b^	-0.23 ^b^	-0.23 ^b^
**Axial / central**									
Pelvis	-0.19	-0.10	-0.10	-0.12	-0.12	-0.13	-0.12	-0.15	-0.14
**Whole body**									
Total body	-0.20 ^b^	-0.12	-0.12	-0.14	-0.14	-0.16	-0.14	-0.15	-0.19
T-score	-0.14	-0.06	-0.06	-0.08	-0.09	-0.10	-0.08	-0.08	-0.09

^a^ Values are Pearson correlation coefficients (r). All coefficients were tested statistically. Because this was an exploratory analysis, no formal correction for multiple comparisons was applied.

^b^ P < 0.05.

^c^ P < 0.01.

## 5. Discussion

This study demonstrated that body composition parameters were associated with BMD in adults with MetS. In this Southeast Asian population, lean mass was positively associated with BMD, whereas central adiposity showed inverse associations with skeletal health.

The LMI and ASMI were significantly and positively correlated with BMD at almost all anatomical sites, consistent with prior studies emphasizing the role of muscle-derived mechanical loading and myokines such as IGF-1 and irisin in promoting osteogenesis (14-16). Another study confirmed that greater muscle mass and strength are associated with higher BMD and reduced fracture risk (14).

The FMI showed weaker and inconsistent correlations with BMD. While some earlier studies proposed that fat mass may confer mechanical benefits for bone,18 recent evidence suggests that excessive adiposity, particularly visceral fat, may be negatively associated with bone metabolism via pro-inflammatory cytokines and altered adipokine signaling (17-19). The android-to-gynoid (A/G) fat ratio, an indicator of central fat distribution, was negatively associated with BMD. This finding was similar to the literature indicating that android fat, rather than general adiposity, is more strongly associated with low BMD and increased fracture risk (10, 17).

Although surgical outcomes were not evaluated in this study, the observed associations between body composition and BMD may have potential relevance in clinical settings where bone quality is an important consideration. DEXA-based assessment may provide additional information on skeletal status and body composition beyond conventional measures such as BMI. In particular, lean mass and fat distribution parameters may be useful in preoperative risk stratification and overall patient evaluation. However, these implications should be interpreted cautiously, and further studies are required to determine whether these associations translate into differences in surgical outcomes.

This study had several limitations. First, the cross-sectional design precludes causal inference. Second, the study population was hospital-based and predominantly female, which may introduce selection bias and limit the generalizability of the findings to men or community-based populations. In addition, the exclusion of participants with prior fracture history may further restrict applicability to older clinical populations in whom fracture history is common.

Third, several potentially important confounders, including menopausal status, physical activity, diabetes severity, calcium intake, and vitamin D status, were not systematically recorded and therefore could not be adjusted for in the analysis. Although BMI is clinically relevant, it was not included as an adjustment variable because of its strong correlation with DEXA-derived body composition parameters, which may introduce multicollinearity.

Fourth, the analysis was limited to unadjusted Pearson correlations without multivariable modeling. Therefore, the observed associations may be related to unmeasured confounding factors and should be interpreted as exploratory rather than independent effects.

Finally, although dual-energy X-ray absorptiometry (DEXA) provides reliable estimates of regional body composition, including android and gynoid fat distribution, it does not directly distinguish visceral from subcutaneous adipose tissue, which may have differential effects on bone metabolism. In addition, bone turnover markers were not assessed, limiting further insight into the underlying biological mechanisms.

### 5.1. Conclusions

In adults with MetS, lean mass was positively associated with BMD, whereas central adiposity, as reflected by the A/G ratio, showed inverse associations with skeletal health, particularly at the spine and long bones. These findings underscore the importance of body composition, beyond BMI alone, in the assessment of bone health in this high-risk population. Dual-energy X-ray absorptiometry-based evaluation provides integrated information on skeletal status and regional body composition that may support clinical assessment. Further prospective and interventional studies are needed to clarify the nature of these relationships and to determine whether modifications in body composition are associated with improvements in skeletal health and clinical outcomes.

## Data Availability

The dataset presented in the study is available on request from the corresponding author during submission or after publication. The data are not publicly available due to our private policy.
